# Feasibility Study of Intelligent Three-Dimensional Accurate Liver Reconstruction Technology Based on MRI Data

**DOI:** 10.3389/fmed.2022.834555

**Published:** 2022-03-17

**Authors:** Shaodong Cao, Huan Li, Suyu Dong, Zhenxuan Gao

**Affiliations:** ^1^Medical Imaging Department of the Fourth Affiliated Hospital of Harbin Medical University, Harbin, China; ^2^The School of Information and Computer Engineering, Northeast Forestry University, Harbin, China; ^3^Neurosurgery Department of the Fourth Affiliated Hospital of Harbin Medical University, Harbin, China

**Keywords:** 3D precise reconstruction technology, liver, cancer, segmentation, simulated liver surgery

## Abstract

Intelligent three-dimensional (3D) reconstruction technology plays an important role in the diagnosis and treatment of diseases. It has been widely used in assisted liver surgery. At present, the 3D reconstruction information of liver is mainly obtained based on CT enhancement data. It has also been commercialized. However, there are few reports on the display of 3D reconstruction information of the liver based on MRI. The purpose of this study is to propose a new idea of intelligent 3D liver reconstruction based on MRI technology and verify its feasibility. Two different liver scanning data (CT and MRI) were selected from the same batch of patients at the same time (patients with a time interval of no more than two weeks and without surgery). The results of liver volume, segmentation, tumor, and simulated surgery based on MRI volume data were compared with those based on CT data. The results show that the results of 3D reconstruction based on MRI data are highly consistent with those based on CT 3D reconstruction. At the same time, in addition to providing the information provided by CT 3D reconstruction, it also has its irreplaceable advantages. For example, multi-phase (early, middle and late arterial, hepatobiliary, etc.) scanning of MRI technology can provide more disease information and display of biliary diseases. In a word, MRI technology can be used for 3D reconstruction of the liver. Hence, a new feasible and effective method to show the liver itself and its disease characteristics is proposed.

## Introduction

With the deepening of medical development and the proposal of personalized and accurate treatment schemes, clinicians are no longer satisfied with the information provided by traditional imaging examination. The promotion of three-dimensional (3D) reconstruction technology greatly meets the needs of clinicians to a certain extent. This technology has been widely used in the human body, especially in hepatobiliary surgery, breast surgery, maxillofacial surgery, and bone surgery (1–3). In 1990, the earliest application in the liver was proposed by Hashimoto et al. (4). Using CT scanning data and computer software, 3D renderings of liver vessels and tumors were reconstructed to guide the surgical treatment of liver cancer. The 3D reconstruction can observe the characteristics of lesions from multiple angles, determine reasonable and effective surgical methods, ensure perioperative safety, and greatly meet the requirements of surgery for accuracy and safety (5). At present, the widely used 3D liver reconstruction technology is based on three-phase CT enhancement to show the anatomical structure of liver parenchyma, important liver vessels and biliary system, and clarify the relationship between liver and tumor and the range of tumor involvement when tumor occurs (6–8). However, some patients choose magnetic resonance liver enhancement examination because of iodine allergy and selective use of magnetic resonance liver-specific contrast agents. Whether these patients can carry out 3D liver reconstruction based on MRI data is a clinical concern. The purpose of this study is to determine the feasibility of accurately obtaining 3D liver reconstruction information based on MRI technology and to explore the clinical significance of 3D reconstruction based on MRI liver enhancement data in guiding clinical selection of accurate and personalized treatment, which plays a positive guiding role in both operation and interventional therapy, so as to reduce complications. Finally, our study provides a new idea for 3D liver reconstruction.

## Data and Methods

### Case Data

Fifty-two patients aged from 31 to 75 years (average age 56.06 years), suspected of liver space occupying, and came to the Fourth Affiliated Hospital of Harbin Medical University from October 2020 to May 2021 were selected as the research subjects. All patients underwent plain CT scan combined with enhancement and plain MRI scan combined with enhancement within one week.

### Inclusion and Exclusion Criteria

Inclusion criteria were as follows: ① All patients received CT and MRI enhancement scanning at the same time within two weeks; ② No corresponding liver treatment was performed between the two examinations; and ③ The image data are relatively complete. Exclusion criteria were as follows: the patient's breath holding was poor, and the image quality affected the diagnosis. All patients signed an informed consent and obtained the approval of the hospital Ethics Committee.

### Scanning Method and Sequence Information

Before examination, the patient was fasted of water for more than 4 h and asked to practice breathing and breath holding to reduced the artifacts caused by respiratory movement as much as possible before being asked to lie on his/her back. The scanning range was from the top of diaphragm to the lower edge of liver. A Canon medical Aquilion ONE 320 slice CT Scanner was used for plain scanning of the liver, gallbladder, spleen, and pancreas. After the plain scanning, a dynamic three-phase enhanced scanning was performed. The contrast agent was administered through the injection of iopanol through elbow vein. The injection rate was 3.5 ml/s, the injection amount was 0.1 mol/kg, and the reconstruction layer thickness was 1 mm. MRI examination was performed with a Philips ingenia 3.0T MRI scanner (Philips Medical Technology) in the Netherlands and an abdominal phased array coil. The scanning sequence was a plain MRI scan, diffusion weighted imaging (DWI) scan, and then a dynamic enhancement scan. The enhancement sequence was performed by an mdixion 3D volume scanning (multi-phase arterial phase, portal phase, delayed phase, and hepatobiliary phase). The contrast agent, an injection amount of 0.1 mol/kg of multihance or promethicin, was injected through an elbow vein at the injection rate of 2 ml/s. Sequence information were as follows: 1) Axial T2WI is TSESPAIR sequence (TR 1000 ms, TE70 ms), imaging field of vision is 300 × 350 × 191 mm, voxel is 1.6 × 1.8 mm, Coronal T2WI is TSE sequence (TR 626 ms, TE 80 ms), imaging field of view is 300 × 402 × 167 mm, voxel is 1.6 × 1.83 mm; 2) Axial DWI (*b* = 800 s/mm2) is sequence with TR 3000 ms and TE 71 ms, imaging field of view is 380 × 297 × 209 mm, voxel is 3 × 3 mm; 3) Axial dynamic enhanced scanning is mdixon-w sequence (TR 3.7 ms, TE 1.31 ms), imaging field of view is 320 × 390 × 225 mm, voxel is 1.75 × 1.76 mm; 4) Coronal enhanced scanning is mdixon-w sequence (TR 5.5 ms, TE 1.34 ms), imaging field of view is 350 × 398 ×150 mm, voxel is 1.7 × 1.7 mm. The layer thickness was 5 mm and the layer spacing was −2.5 mm. The contrast agent was injected with MODIS or promethicin through elbow vein. The injection rate was 2 ml/s and the injection volume was 0.1 mol/kg.

### Image Analysis and Post-Processing

Computed tomography (CT) and MRI scanning images were evaluated by two experienced senior doctors. When the evaluation of the two doctors were inconsistent, the image rating was carried out through negotiation. Since liver segmentation and simulated surgery are based on the portal and hepatic vein, the development of portal vein is basically good. This is because the development quality of hepatic vein directly determines the accuracy of 3D liver reconstruction. Therefore, one radiologist (observer 1) is familiar with abdominal imaging diagnosis and one hepatobiliary surgeon (observer 2) are responsible for Hepatic veins (HVS) in each group. The roughest Hepatic vein (HV) in the image was evaluated independently. The scoring criteria were as follows: no display of HVS was recorded as 0 points; blurred, clear, and sharp pipe wall were recorded as 1, 2, and 3 points, respectively; and slightly higher, higher, and significantly higher lumen density were recorded as 1 point, 2 points, and 3 points, respectively. The scores were divided into three levels: excellent (5–6 points), average (3–4 points), and poor (0–2 points).

The qualified image is transferred to the Philips Nebula workstation and the surface rendering algorithm was automatically used for 3D reconstruction. The liver volume and the left and right lobes of the liver are represented based on 3D reconstruction. The measurement of volume for each liver was repeated by three times, in which various values are recorded, and the average value is taken.

## 3D Liver Model Reconstruction and Measurement

The digitized liver model obtained by CT 3D reconstruction and the fourth level portal vein vessels were clearly displayed. The original data of plain CT scan phase, arterial phase, portal phase, and delayed phase are imported into the Philips Nebula workstation (Philips Medical Technology) to display the information of transverse axis, sagittal position, and coronal position of the original data.

### The Liver Reconstruction and Measurement Based on CT Image

**Figure 1:**
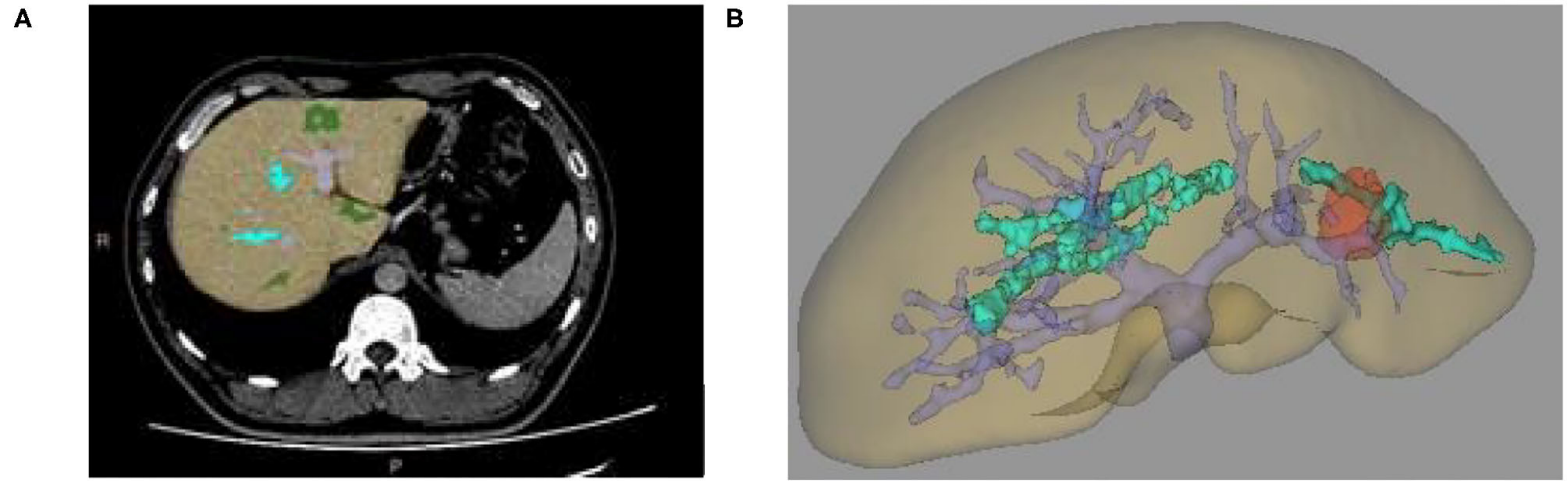
**(A)** Two-dimensional CT enhanced image, **(B)** Three-dimensional (3D) reconstruction of liver and tumor based on growth model on CT data.

**Figure 2:**
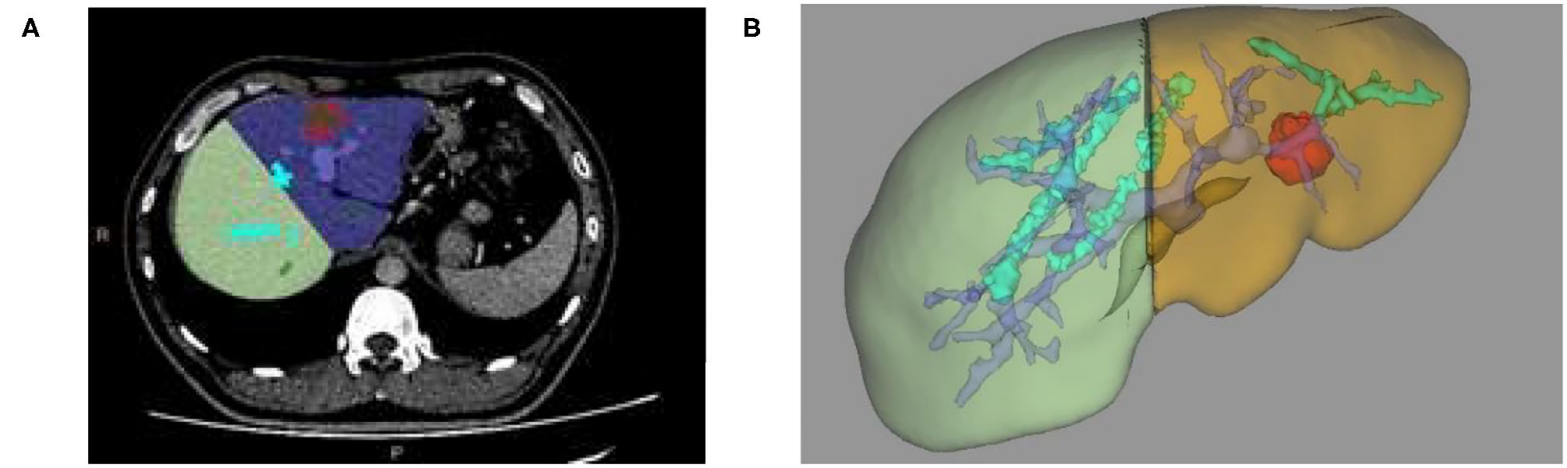
**(A)** Two-dimensional CT enhanced image, **(B)** 3D reconstruction based on liver segmentation and tumor growth model on CT data.

The liver model is established by using the 3D reconstruction organ cutting method based on portal phase volume data. Then, the volume data on liver CT-enhanced portal phase was reconstructed based on Marching Cube surface rendering algorithm:

(1) We automatically reconstructed the liver model according to the liver position and CT value based on the surface rendering algorithm. At the same time, according to the difference between vascular CT value and liver CT value, the portal vein and hepatic vein were automatically segmented and different sequences (coronal, sagittal, and transverse axial) are compared. Whether the automatic selection contained the liver model was checked. If the automatic segmentation was wrong, several seed points were manually constructed and the threshold range was appropriately adjusted to induce all seed points to fill and grow to contain liver tissue and eliminate non-liver tissue. Finally, a standard liver model was made (Figure 1A).
(2) At the same time, according to the difference of CT values, seed points were manually constructed to fill the growing tumor model (Figure 1B).
(3) According to the position of blood vessels such as portal vein and hepatic vein, the liver is automatically segmented to obtain the liver volume of each segment of the left and right lobes. At the same time, simulated hepatectomy is carried out according to clinical needs to calculate the residual liver volume to determine whether surgical treatment can be carried out directly in a clinic (as shown in Figures 2A,B).

### Liver Reconstruction and Measurement Based on MRI Image

**Figure 3:**
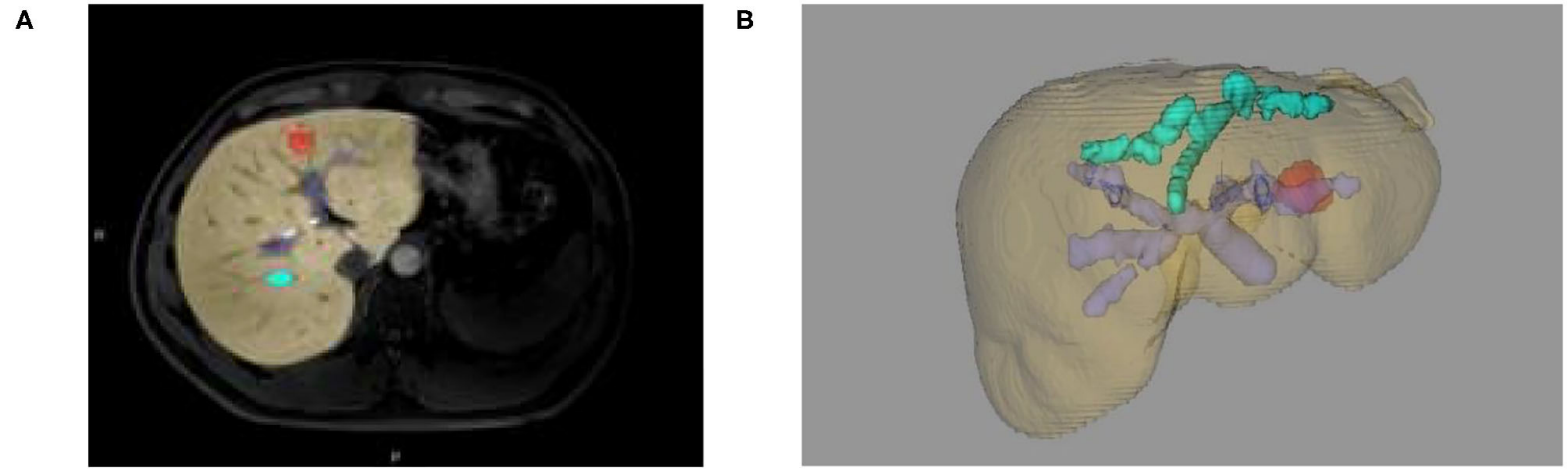
**(A)** Two-dimensional MRI enhanced image, **(B)** 3D reconstruction of liver and tumor based on growth model on MRI data.

**Figure 4:**
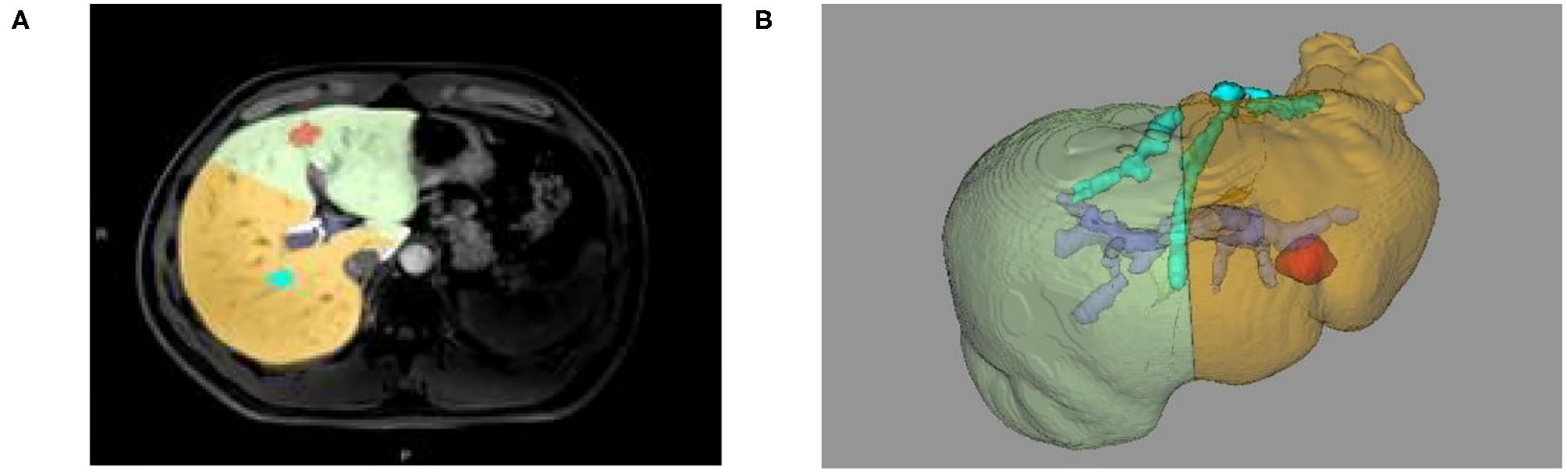
**(A)** Two-dimensional MRI enhanced image, **(B)** 3D reconstruction based on liver segmentation and tumor growth model on MRI data.

The establishment of the liver model was based on the 3D reconstruction organ cutting method based on the volume data of the portal vein phase (the signal of portal vein and hepatic vein is higher than the signal of the liver parenchyma) or hepatobiliary phase (the signal of portal vein and hepatic vein is lower than the signal of the liver parenchyma). The liver MRI volume data was reconstructed based on the Marching Cube surface-rendering algorithm from Philips Nebula workstation. Since the MRI data cannot be directly transferred into the liver post-processing software, the volume data was first transferred into the AVA vascular processing software. Only then Was the secondary post-processing software selected to enter the liver software. Because the liver in the MRI data cannot be automatically identified, it is manually identified as follows:

(1) Several seed points were manually constructed on the liver. After the threshold range was properly adjusted, all seed points were induced to fill the growing liver tissue. After comparing the data from different sequences (coronal position, sagittal position, and transverse axis position), we analyzed whether the seed points contained the liver model. Then, we reconstructed the 3D liver model based on Marching Cube algorithm to make the liver model standard and complete (as shown in Figure 3A);
(2) The reconstruction of the 3D models of liver space of which the portal vein, hepatic vein, and liver segmentation occupy is realized. Likewise, simulated liver surgery, via the same method in liver reconstruction and measurement based on CT image, is made possible (as shown in Figures 3B, 4A,B).

### Statistical Analysis

The total liver volume, right liver volume, and the clarity of hepatic vein from the two groups of patients were measured at the same time and compared between the two groups. The SPSS24.0 software was used for data analysis. The counting data is expressed as n. The data measurement was based on the normal distribution with parameters X ± S index. Two independent samples were analyzed by univariate analysis using *t*-test, *p* < 0.05 indicated that there is a statistical significance.

## Results

All 52 patients were divided into a CT group and an MRI group according to the scanning method. We compared and analyzed the data from the two groups on the aspect of functional volume, total volume, right liver volume, and hepatic vein score. It can clearly be seen that the 3D reconstruction based on MRI volume data is consistent with the 3D reconstruction results based on CT volume data (*p* > *0*.05) (Tables 1, 2). Moreover, the clarity of hepatic vein display in MRI group was significantly better than that in CT group (*p*<*0*.05) (as shown in Table 3).

**Table 1:** Liver volume measured by CT and MRI reconstruction.

**Project**	**CT group**	**MRI group**	**Inspection value**	***P* value**
Functional volume (cm^3^, $\bar{x}$ ± s)	1270.5 ± 308.6	1257.0 ± 314.0	−0.739	0.464
Total volume (cm^3^, $\bar{x}$ ± s)	1305.8 ± 315.0	1284.4 ± 323.4	−1.145	0.257

**Table 2:** Right lobe volume measured by CT and MRI reconstruction.

**Project**	**CT group**	**MRI group**	**Inspection value**	***P* value**
Right liver volume (cm^3^, $\bar{x}$ ± s)	824.6 ± 246.9	826.2 ± 248.3	0.110	0.913

**Table 3:** Clarity analysis of hepatic vein reconstructed by CT and MRI.

**Project**	**CT group**	**MRI group**	***T*-test**	***P* value**
Hepatic vein score [score, M (P_25_,P_75_)]	4 (2~6)	5 (5~6)	−3.963	<0.01

## Discussion

### The Necessity of Selecting 3D Reconstruction Technology Based on MRI Data

It is well-known that MRI has certain advantages over CT. Firstly, MRI is non-invasive and non-radiographic and hence will not bring biological damage. Moreover, MRI has advantages in sensitivity and spatial resolution, which is conducive to the comparative observation of soft tissue (9). On the other hand, some people are allergic to iodine contrast agent in CT examination and passively give up CT examination. Also, some patients directly choose a liver-specific MRI contrast agent for enhancement in physical examination ultrasound, while other patients only choose MRI examination due to various concerns and refuse CT examination. Gadolinium-DTPA (GD-DTPA) is the most commonly used contrast agent for liver MRI enhancement, while hepatobiliary MRI can provide more lesion details and better qualitative analysis of disease characteristics. The most widely used clinical contrast agents are gadolinium ethoxybenzyl diethylenetria mine pentaacetic acid (GD-EOB-DTPA) whose trade names are prometaxel and gadobenate dimeglumine (GD-BOPTA). It is found (10, 11) that both MODIS and promethicin can be ingested by hepatocytes and excreted into the biliary system, which can significantly improve the sensitivity and specificity of related liver cancer. They are mainly used for the diagnosis of focal lesions in the liver, especially for the detection of small lesions and the identification of benign and malignant nodules (12). In addition, some literatures suggest that the uptake of GD-EOB-DTPA by hepatocytes in patients with liver cirrhosis is delayed. Therefore, it can also be used for the staging of liver fibrosis and the non-invasive evaluation of liver function. With the clinical popularization of hepatobiliary specific contrast agents, enhanced MRI has more advantages in the detection of early liver cancer. Without the assistance of MRI 3D reconstruction technology, it is difficult to the accurate preoperative evaluation and simulated operation to a certain extent. Does it mean that the assistance of 3D reconstruction is lost without the addition of CT examination? The results of this study prove that 3D reconstruction technology based on MRI enhancement data is feasible (13).

### Advantages of MRI 3D Reconstruction in Analyzing Liver Segmentation

Computed tomography (CT) 3D reconstruction is based on the portal phase. Through the display of hepatic vein and portal vein, the four-level vascular supply area of the liver is analyzed and segmented. According to the results of this experiment, the clarity of the hepatic vein in the MRI group was significantly better than that in CT group (*p* < 0.05). In addition, accurate 3D reconstruction technology based on MRI can not only be based on portal phase, but also refer to hepatobiliary phase. Compared with CT portal phase images, MRI has certain advantages in terms of vascular imaging uniformity and contrast with liver parenchyma. MRI makes the clear display of biliary system in hepatobiliary phase after using hepatobiliary specific contrast agent—a display which cannot be provided by CT data. Conventional magnetic resonance cholangiopancreatography shows the morphological characteristics of bile duct tree and pancreatic duct by inhibiting the soft tissue and bone in all backgrounds. It cannot reflect the functional characteristics of bile duct. Therefore, some patients with ascites will affect its display. Under the action of hepatobiliary-specific contrast agents, cholangiography can be clearly displayed by T1-weighted images. MRI cholangiography, which is widely studied at present, is very helpful for the localization and qualitative diagnosis of biliary diseases. Hepatobiliary-specific contrast-enhanced MRI has become a new method to evaluate liver function (14). Hepatobiliary phase images using hepatobiliary-specific magnetic resonance contrast agents can provide more detailed information of liver lesions, including qualitative analysis of lesions and analysis of hepatocyte function (15). This can not only evaluate liver function and reserve function, but also predict postoperative functional liver volume more accurately combined with 3D reconstruction technology.

### Analysis of Liver Volume in 3D Reconstruction Technology of CT and MRI

The results of this experimental study show that there is no significant difference between MRI 3D reconstruction technology and CT 3D reconstruction technology. From the MR 3D reconstruction image, we can also see that the boundary of the liver is slightly blurred compared with the edge of the CT 3D reconstruction model. Hence, it may slightly expand the resection range of liver lesions to a certain extent. This presentation of blurred boundaries may be related to the layer thickness of MRI liver scanning. Therefore, the relevant influencing factors are expected to be corrected by adjusting the layer thickness and other parameters in a follow-up study. However, MRI has many parameters and contains a large amount of information. The combination of multiple sequences and the application of special imaging parameters has important research value and research potential. Many patients with liver cancer have a history of fatty liver. The comparison of the same inverse bitmap can help the qualitative diagnosis of fatty liver. Moreover, the application of quantitative magnetic susceptibility weighted map sequence makes it possible to quantitatively diagnose liver fat deposition and clarify the abandoned functional liver volume with different texture and reserve function to more accurately and effectively calculate the functional liver volume. Hence, reducing the incidence of postoperative liver failure.

### The Significance of 3D Reconstruction Technology of CT and MRI for Surgery

Many patients with primary liver cancer are complicated with hepatitis and cirrhosis. Liver failure may occur after hepatectomy, resulting in a low expression of hepatocyte growth factor, a reduction of the volume of liver regeneration, and an increase of the risk of postoperative mortality in patients with primary liver cancer (16). Liver failure after hepatectomy is the main cause of death of perioperative patients. The main reason is that the residual liver function is not enough to meet the needs of human body (17). Therefore, it is very important to accurately evaluate the liver reserve function beforehand. According to the experimental results, the results of 3D reconstruction technology of whole liver volume and right liver volume are consistent. Since the outline of MRI data is based on points, the total volume and segmented volume data are consistent. It can be seen that the measurement of other volumes is also consistent. According to the current research (7, 18–20), CT 3D reconstruction technology can accurately predict the scope of hepatectomy, evaluate the residual liver volume by simulating the residual liver volume after hepatectomy, evaluate the liver reserve function, guide the formulation of surgical scheme and assist in the discrimination of complex surgery so as to accurately judge the prognosis and outcome of liver surgery, and give full play to the advantages and characteristics of surgery. Therefore, the accuracy of 3D reconstruction simulation surgery based on MRI data is of great clinical value.

## Deficiencies and Prospects

This study is a retrospective study. Magnetic Resonance (MR) data are conventional data without prospective design and optimization. In the next study, the impact of scanning parameter optimization on 3D reconstruction model can be studied. In addition, all 3D reconstruction models based on MRI are manually sketched by using the 3D post-processing software of CT. This brings some inconvenience and factors to the operation process. A study on effective semi-automatic or even full-automatic 3D post-processing method based on plain MRI scan and hepatobiliary phase may further assist the application of clinical work.

## Conclusion

It is feasible to accurately obtain the 3D reconstruction information of liver based on MRI technology. In addition, the 3D reconstruction based on MRI data has its irreplaceable advantages in addition to the information provided by CT 3D reconstruction.

## Data Availability Statement

The raw data supporting the conclusions of this article will be made available by the authors, without undue reservation.

## Ethics Statement

The studies involving human participants were reviewed and approved by Ethics Committee of the Fourth Affiliated Hospital of Harbin Medical University. The patients/participants provided their written informed consent to participate in this study. Written informed consent was obtained from the individual(s) for the publication of any potentially identifiable images or data included in this article.

## Author Contributions

SC and HL conceived and designed most of the study. SD performed the simulations, analyses, and wrote most of the manuscript. ZG contributed to the figure design and manuscript writing. SC supervised the project. All authors contributed to the article and approved the submitted version.

## Funding

This work was supported by the Horizontal Project: The Application of Image Data Quality Control in 3D Printing Technology, Project No. 2020-19, by China Postdoctoral Science Foundation under Grant 2021M690574, and by Interdisciplinary Research Foundation of HIT under Grant IR2021230.

## Conflict of Interest

The authors declare that the research was conducted in the absence of any commercial or financial relationships that could be construed as a potential conflict of interest.

## Publisher's Note

All claims expressed in this article are solely those of the authors and do not necessarily represent those of their affiliated organizations, or those of the publisher, the editors and the reviewers. Any product that may be evaluated in this article, or claim that may be made by its manufacturer, is not guaranteed or endorsed by the publisher.

## References

[1] Smelt J Pontiki A Jahangiri M Rhode K Nair A Bille A. 3D printing for chest wall reconstruction in thoracic surgery: building on experience. Thorac Cardiovasc Surg. (2020) 68:352–6. 10.1055/s-0039-1678611 30736084

[2] Breik O Idle M Martin T Praveen P Parmar S. 3D computer-assisted surgical planning and manufacturing in complex maxillary reconstruction. Atlas Oral Maxillofac Surg Clin North Am. (2020) 28:151–64. 10.1016/j.cxom.2020.05.008 32741512

[3] Mehta S Byrne N Karunanithy N Farhadi J. 3D printing provides unrivalled bespoke teaching tools for autologous free flap breast reconstruction. J Plast Reconstr Aesthet Surg. (2016) 69:578–80. 10.1016/j.bjps.2015.12.026 26906554

[4] Hashimoto D Dohi T Tsuzuki M Horiuchi T Ohta Y Chinzei K. Development of a computer-aided surgery system: 3D graphic reconstruction for treatment of liver cancer. Surgery. (1991) 109:589–96. 1850556

[5] Yamanaka J Okada T Saito S Kondo Y Yoshida Y Suzumura K. Minimally invasive laparoscopic liver resection: 3D MDCT simulation for preoperative planning. J Hepatobiliary Pancreat Surg. (2009) 16:808–15. 10.1007/s00534-009-0112-8 19466379

[6] Cai W Fan Y Hu H Xiang N Fang C Jia F. Postoperative liver volume was accurately predicted by a medical image three dimensional visualization system in hepatectomy for liver cancer. Surg Oncol. (2017) 26:188–94. 10.1016/j.suronc.2017.03.006 28577725

[7] He YB Bai L Jiang Y Ji XW Tai QW Zhao JM. Application of a 3D reconstruction technique in liver autotransplantation for end-stage hepatic alveolar echinococcosis. J Gastrointest Surg. (2015) 19:1457–65. 10.1007/s11605-015-2842-z 25967139

[8] Chinese SODM Liver CCOCMDA Clinical PMCOCMDA Digital ISCOCRHA. [Clinical practice guidelines for precision diagnosis and treatment of complex liver tumor guided by 3D visualization technology (version 2019)]. Nan Fang Yi Ke Da Xue Xue Bao. (2020) 40:297–307. 10.12122/j.issn.1673-4254.2020.03.01 32376594 PMC7167330

[9] Usman S Smith L Brown N Major V. Diagnostic accuracy of magnetic resonance imaging using liver tissue specific contrast agents and contrast enhanced multi detector computed tomography: a systematic review of diagnostic test in hepatocellular carcinoma (HCC). Radiography (Lond). (2018) 24:e109–14. 10.1016/j.radi.2018.05.002 30292515

[10] Li XQ Wang X Zhao DW Sun J Liu JJ Lin DD. Application of Gd-EOB-DTPA-enhanced magnetic resonance imaging (MRI) in hepatocellular carcinoma. World J Surg Oncol. (2020) 18:219. 10.1186/s12957-020-01996-4 32828123 PMC7443289

[11] Lebert P Adens-Fauquembergue M Azahaf M Gnemmi V Behal H Luciani A. MRI for characterization of benign hepatocellular tumors on hepatobiliary phase: the added value of in-phase imaging and lesion-to-liver visual signal intensity ratio. Eur Radiol. (2019) 29:5742–51. 10.1007/s00330-019-06210-y 30993437

[12] Kierans AS Kang SK Rosenkrantz AB. The diagnostic performance of dynamic contrast-enhanced MR imaging for detection of small hepatocellular carcinoma measuring up to 2 cm: a meta-analysis. Radiology. (2016) 278:82–94. 10.1148/radiol.2015150177 26098460

[13] Wu JW Yu YC Qu XL Zhang Y Gao H. Optimization of hepatobiliary phase delay time of Gd-EOB-DTPA enhanced magnetic resonance imaging for identification of hepatocellular carcinoma in patients with cirrhosis of different degrees of severity. World J Gastroenterol. (2018) 24:415–23. 10.3748/wjg.v24.i3.415 29391764 PMC5776403

[14] Yoon JH Lee JM Kang HJ Ahn SJ Yang H Kim E. Quantitative assessment of liver function by using gadoxetic acid-enhanced MRI: hepatocyte uptake ratio. Radiology. (2019) 290:125–33. 10.1148/radiol.2018180753 30375932

[15] Li XM Chen Z Xiao EH Shang QL Ma C. Diagnostic value of gadobenate dimeglumine-enhanced hepatocyte-phase magnetic resonance imaging in evaluating hepatic fibrosis and hepatitis. World J Gastroenterol. (2017) 23:3133–41. 10.3748/wjg.v23.i17.3133 28533670 PMC5423050

[16] Ma JL He LL Li P Jiang Y Hu JL Zhou YL Liang XX. Clinical features and outcomes of repeated endoscopic therapy for esophagogastric variceal hemorrhage in cirrhotic patients: ten-year real-world analysis. Gastroenterol Res Pract. (2020) 2020:5747563. 10.1155/2020/5747563 32508912 PMC7245665

[17] Wang YY Zhong JH Su ZY Huang JF Lu SD Xiang BD. Albumin-bilirubin versus Child-Pugh score as a predictor of outcome after liver resection for hepatocellular carcinoma. Br J Surg. (2016) 103:725–34. 10.1002/bjs.10095 27005482

[18] Zhang J Qiao QL Guo XC Zhao JX. Application of 3D visualization technique in preoperative planning of progressive hilar cholangiocarcinoma. Am J Transl Res. (2018) 10:1730–5. 30018714 PMC6038071

[19] Wang P Que W Zhang M Dai X Yu K Wang C. Application of 3-dimensional printing in pediatric living donor liver transplantation: a single- center experience. Liver Transpl. (2019) 25:831–40. 10.1002/lt.25435 30770639

[20] Wu TC Fang CH Liu WY Cai W Fan YF Yang J. 3D reconstruction aids surgery for complicated hepatolithiasis. Hepatogastroenterology. (2014) 61:613–22. 26176045

